# Identity and mechanisms of alkane-oxidizing metalloenzymes from deep-sea hydrothermal vents

**DOI:** 10.3389/fmicb.2013.00109

**Published:** 2013-05-10

**Authors:** Erin M. Bertrand, Ramaydalis Keddis, John T. Groves, Costantino Vetriani, Rachel Narehood Austin

**Affiliations:** ^1^Department of Chemistry, Bates CollegeLewiston, ME, USA; ^2^Microbial and Environmental Genomics, J. Craig Venter InstituteSan Diego, CA, USA; ^3^Department of Biochemistry and Microbiology, Rutgers UniversityNew Brunswick, NJ, USA; ^4^Institute of Marine and Coastal Sciences, Rutgers UniversityNew Brunswick, NJ, USA; ^5^Department of Chemistry, Princeton UniversityPrinceton, NJ, USA

**Keywords:** alkanotrophs, hydrocarbon oxidation, deep-sea hydrothermal vents, alkanes, alkane hydroxylases

## Abstract

Six aerobic alkanotrophs (organism that can metabolize alkanes as their sole carbon source) isolated from deep-sea hydrothermal vents were characterized using the radical clock substrate norcarane to determine the metalloenzyme and reaction mechanism used to oxidize alkanes. The organisms studied were *Alcanivorax* sp. strains EPR7 and MAR14, *Marinobacter* sp. strain EPR21, *Nocardioides* sp. strains EPR26w, EPR28w, and *Parvibaculum hydrocarbonoclasticum* strain EPR92. Each organism was able to grow on *n*-alkanes as the sole carbon source and therefore must express genes encoding an alkane-oxidizing enzyme. Results from the oxidation of the radical-clock diagnostic substrate norcarane demonstrated that five of the six organisms (EPR7, MAR14, EPR21, EPR26w, and EPR28w) used an alkane hydroxylase functionally similar to AlkB to catalyze the oxidation of medium-chain alkanes, while the sixth organism (EPR92) used an alkane-oxidizing cytochrome P450 (CYP)-like protein to catalyze the oxidation. DNA sequencing indicated that EPR7 and EPR21 possess genes encoding AlkB proteins, while sequencing results from EPR92 confirmed the presence of a gene encoding CYP-like alkane hydroxylase, consistent with the results from the norcarane experiments.

## Introduction

The microbial transformation of alkanes, saturated energy-rich hydrocarbons, is significant for a number of reasons (Das and Chandran, 2011). Alkanes are a major constituent of petroleum and natural gas and are a primary energy source for human society. Alkanes are toxic to some microorganisms and a source of energy and carbon for others, including, perhaps, ancient organisms from which life arose (Kobayashi and Yanagawa, 2002). The selective activation of carbon-hydrogen bonds, necessary for microbial alkane transformation, is one of the most energetically difficult processes accomplished in nature (Borovik, 2011). Understanding biological alkane activation could yield insight into new methods for industrial production of valuable materials and improve our understanding of molecular level processes underpinning the global carbon cycle (Bosetti et al., 1992; Shilov and Shul'pin, 1997; Baik et al., 2003; Groves, 2006; Que and Tolman, 2008).

Alkanes originate from a variety of sources, the largest of which are biogenic (McCollom, 2013). These biogenic sources include thermogenesis, the geochemical processing of decaying plant and algal matter, as well as direct production by living organisms (Volkman, 2006). There are also abiotic sources of alkanes (Parson et al., 1995; Sherwood Lollar et al., 2002). Methane and other low molecular weight alkanes are produced abiogenically through water-rock interactions under conditions of high temperature and pressure and are discharged through fractures in places like hard rock mines and oceanic thermal vents (Lancet and Anders, 1970; Parson et al., 1995; Sherwood Lollar et al., 2002). In hydrothermal systems, abiotic synthesis of hydrocarbons may involve Fisher-Tropsch reactions and the serpentinization of ultramafic rocks (Lancet and Anders, 1970; Berndt et al., 1996; McCollom et al., 1999). In general, abiotic synthesis is thought to contribute relatively small amounts of alkanes to the global stock, though it may be a significant source in geothermal environments (Sherwood Lollar et al., 2002; Proskurowski et al., 2008). There have been some suggestions that such abiogenically produced hydrocarbons could have played a role in the origin and evolution of early life (Martin et al., 2008).

Alkane oxidation has evolved in a variety of organisms both as a strategy for hydrocarbon detoxification and to harness the carbon and energy stored in alkanes (Hanson and Thomas, 1996; Van Beilen et al., 2003; Van Beilen and Enrico, 2005; Hakemian and Rosenzweig, 2007). Hydrocarbonoclastic bacteria are those that can utilize hydrocarbons as their sole source of carbon and energy. In surface waters, they have been identified as members of the genera *Alcanivorax, Marinobacter, Cycloclasticus, Neptunomonas, Oleiphilus, Oleispira*, and *Planococcus* (Harayama et al., 1999; Yakimov et al., 2004; Wang and Shao, 2012b; Viggor et al., 2013). Members of the *Parvibaculum* species may be involved in the global cycling of alkanes, as well as other anthropogenic pollutants (Schleheck et al., 2011). Though aerobic alkane metabolism involves many steps (Van Beilen et al., 2003) the initial oxidation, which involves breaking a strong non-polar C–H bond, is the chemically most challenging and intriguing (Marquez-Rocha et al., 2005; Nakano et al., 2011; Al-Awadhi et al., 2012; Sun et al., 2012).

Eight different families of aerobic alkane-oxidizing enzymes have been identified in marine bacteria (Austin and Groves, 2011). Methane is oxidized primarily by particulate methane monooxygenase (pMMO), encoded by the *pmo* genes (Hanson and Thomas, 1996; Hakemian and Rosenzweig, 2007; Rosenzweig, 2011) although soluble methane monooygenase (sMMO) has been shown to be functional in a few instances (Hakemian and Rosenzweig, 2007). Propane (pMO) and butane monooxygenases (bMO) have both been identified in microorganisms that can metabolize short chain alkanes (Sluis et al., 2002; Arp et al., 2007; Cooley et al., 2009; Redmond et al., 2010). Medium chain (C_5_–C_22_) alkanes are oxidized by particulate alkane hydroxylases (pAHs, e.g., AlkB) and/or cytochrome P450/CYP153 enzymes (Smits et al., 1999, 2002; Van Beilen et al., 2006; Van Beilen and Funhoff, 2007). Recently, a flavin-binding monoxygenase, encoded by the *almA* gene, has been shown to be involved in the metabolism of long chain *n*-alkanes of C_32_ and longer (Throne-Holst et al., 2006, 2007; Wang and Shao, 2012a). Another flavin-binding monooxygenase, LadA, has also been identified (Li et al., 2008). Information about the active site structures of these enzymes and their presumed mechanisms has been recently reviewed (Austin and Groves, 2011).

Though biotic alkane hydroxylation occurs in a wide range of environments (Das and Chandran, 2011), the implications of alkane hydroxylase activity in deep-sea hydrothermal vents are of particular interest. Deep-sea hydrothermal vents are found at sites of seafloor spreading at mid-ocean ridges as well as at other tectonically active regions, such as hot spot volcanoes and back-arc spreading centers (Simoneit, 1993; Van Dover, 2000). Some hydrothermal vent fluids contain high to moderate amounts of hydrocarbons, including *n*-alkanes (Whelan and Hunt, 1983; Whelan et al., 1988; Simoneit, 1993; Higashihara et al., 1997). For instance, medium-chain *n*-alkanes (between *n*-C_13_ and *n*-C_21_), were found to be 100 to 400 times more concentrated in warm fluids venting from fissures in basalt on the East Pacific Rise (EPR) than in the surrounding seawater (Brault et al., 1988). Furthermore, more recent studies revealed the presence of hydrocarbons derived from thermogenic processes in higher temperature portions of the subsurface reaction zone in geothermal systems off the coast of New Zealand and on the Juan de Fuca Ridge (Botz et al., 2002; Cruse and Seewald, 2006).

These hydrothermal fluids are generally highly reducing and anoxic, but when they mix with colder, oxygenated water, steep thermal and redox gradients are generated (Perner et al., 2012). These gradients are exploited by microbes and support complex ecosystems surrounding hydrothermal vents. The oxidation of methane and other short chain alkanes is an important metabolism in vent environments and has been shown to aid in supporting higher trophic levels (Fujiwara et al., 2000). The anaerobic oxidation of methane in vent sediments it has been shown to be a powerful driver of carbon cycling (Wankel et al., 2012). Sulfate-reducing bacteria from sediments in the Gulf of Mexico and Guaymas Basin have been shown to anaerobically oxidize short alkanes (Kniemeyer et al., 2007). Additionally, *pmo* transcripts were found to be abundant in vent plumes and deep water sites, suggesting that methane oxidation is important in the aerated water column as well (Lesniewski et al., 2012). However, while organisms that oxidize mid or long -chain (>C_6_) alkanes have been isolated from hydrothermal vent environments, including both plume water and sediments, our knowledge of the diversity and ecological role of >C_6_ alkane oxidizers in these environments remains limited (Bazylinski et al., 1989; Rosario-Passapera et al., 2012). Given that marine microbes are less well-characterized than their terrestrial counterparts, it is possible that new enzymes and possibly even new chemical approaches to converting alkanes to alcohols may be found in vent environments (Harayama et al., 1999; Harayama and Hara, 2004).

In this paper we examine the reaction mechanisms of alkane hydroxylation in six different bacteria isolated from deep-sea vents that were able to grow on mid-chain (C_8_–C_16_) alkanes as their sole carbon source, using a diagnostic substrate, bicyclo[4.1.0]heptane (norcarane). Enzymatic oxidation of norcarane generates different products depending on the reaction mechanism employed by the enzyme. As such, norcarane oxidation studies are able to functionally distinguish between AlkB and cytochromes P450 (CYP) in purified enzymes as well as in whole-cell bioassays and it is described as a “diagnostic substrate” for this reason (Rozhkova-Novosad et al., 2007).

Norcarane can be hydroxylated in three different ways; each creates a different distribution of oxidized products (Austin et al., 2000). Homolytic bond cleavage creates a substrate-based radical intermediate, while heterolytic bond cleavage creates a carbon-centered cationic intermediate. When homolytic bond cleavage at the carbon α to the cyclopropyl group occurs, a carbon radical is generated. This enables the three-membered carbon ring to open, relieving ring strain. The norcarane ring opens with an internal rearrangement rate of 2 × 10^8^ s^−1^ (Austin et al., 2000, 2006). Internal molecular rearrangement occurs while the enzyme is carrying out the next step in the catalytic cycle, the “rebound step” where “OH.” combines with the substrate radical to form an alcohol. The rate of this enzyme-specific step is termed the “rebound rate.” The rebound rate can be calculated from the ratio of the concentration of unrearranged alcohol products (at the 2-position only) generated during oxidation to the concentration of rearranged products multiplied by the intramolecular rearrangement rate for the molecule itself (Equation 1). The average lifetime of the substrate radical is given by the reciprocal of the rebound rate. Using this approach, norcarane has yielded valuable information about enzyme active site structure and function for cytochrome P450 enzymes (Auclair et al., 2002), alkane monooxygenase AlkB (Austin et al., 2000; Cooper et al., 2012; Naing et al., 2013), xylene monooxygenase (Austin et al., 2003), toluene monooxygenase (Moe et al., 2004) as well as soluble methane monooxygenase (Brazeau et al., 2001).

Equation 1. Enzyme rebound rate constant and radical lifetime.

$$\begin{array}{l} \;k_{\text{rebound}\;\text{rate}\;\text{constant}}=k_{\text{rearrangement}}\left(\frac{\left[\text{ring closed}\right]}{\left[\text{ring opened}\right]}\right)\\ \text{radical lifetime}=\frac{1}{k_{\text{rebound}}} \end{array}$$

In this work, we hypothesized that norcarane would be a substrate for these novel organisms and that identifying the products formed from its transformation would provide a means to provisionally identify the enzymes being used for alkane oxidation by vent bacteria. Our results show that both CYP and AlkB-like enzymes are functional proteins that contribute to alkane-oxidation in deep-sea hydrothermal vent bacterial isolates, suggesting that these metalloenzymes may be of importance in hydrothermal vent ecosystems.

## Materials and methods

### Chemicals

Chemicals and solvents were purchased from Sigma–Aldrich Corp. (St. Louis, MO) or BioRad (Hercules, CA). The diagnostic substrate bicyclo [4.1.0] heptane (norcarane), was synthesized and purified following published procedures (Smith and Simmons, 1973). Product standards were characterized on a Bruker Advance™ 400 MHz Nuclear Magnetic Resonance (NMR) Spectrometer at ambient temperature.

### Organisms

Hydrothermal fluids were collected from diffuse flow deep-sea vents located on the EPR and the Mid-Atlantic Ridge (MAR). The fluids were collected using titanium samplers operated by the manipulator of the Deep-Submergence Vehicle *Alvin*. In the laboratory, 1 ml aliquots of fluid were inoculated into 10 ml of Artificial Sea Water Minimal Medium (ASW MM) (Crespo-Medina et al., 2009). Each tube was then supplemented with dodecane (C_12_H_26_) in the vapor phase as the only carbon and energy source and was incubated at various temperatures (Table 1). Pure cultures were isolated by successive transfers of single colonies on ASW MM/dodecane solidified with Noble agar (Sigma). Once the isolation of pure cultures was completed, the ability of the isolates to grow in complex Artificial Sea Water Medium (ASW; l^−1^: NaCl, 24 g; KCl, 0.7 g; MgCl, 7.0 g, yeast extract, 3.0 g; peptone, 2.5 g) was tested. All six hydrothermal vent isolates were able to grow in complex ASW medium. However, for the purpose of the experiments described here, the six hydrothermal vent isolates (EPR7, EPR21, EPR26w, EPR28w, MAR14, EPR92) were grown aerobically on alkanes as the sole carbon source in ASW MM at the appropriate temperature (Table 1) at 300 rpm for up to 1 week, subcultured into fresh media and then were allowed to grow to an optical density (OD) near 0.1. The diagnostic substrate was then introduced in the vapor phase (30–50 μL in 50 mL) to the growing cultures via a hanging bulb apparatus, as previously described (Bertrand et al., 2005). An OD of 0.1 was chosen for metabolic analysis because cultures at this density were in exponential growth phase (data not shown). The cultures were incubated at their appropriate temperatures at 300 rpm for 4–18 h.

**Table 1:** **Characteristics of hydrothermal vent isolates**.

**Isolate**	**EPR7**	**EPR21**	**ERP26w, 28w**	**MAR14**	**EPR92**
Temp range (°C)	37–45	28–37	28–30	28–30	20–40
Genus of closest characterized relative (16S rRNA gene accession No; Sequence identity)	*Alcanivorax dieselolei* (EF647617; 99%)	*Marinobacter* sp. (AY196982; 99%)	*Nocardioides* sp. (HM222686; 99%)	*Alcanivorax borkumensis* (NR074890; 99%)	*Parvibaculum hydrocarbonoclasticum* DSM 23209^a^, (GU574708; 100%)
Type of sample, vent site, location	Diffuse flow vent, Mk119, East Pacific Rise (EPR), 9° N, 104° W	Diffuse flow vent, Mk119, EPR, 9° N, 104° W	Diffuse flow vent, Mk119, EPR, 9° N, 104° W	Diffuse flow vent, Lucky Strike, Mid-Atlantic Ridge (MAR), 37° N, 32° W	Diffuse flow vent EPR, Tica, 9° N, 104° W
Collection date	May 1999	May 1999	May 1999	July 2001	April 2004
Depth of vent site	2500 m	2500 m, 1 m above source	2500 m, 1 m above source	1700 m	2513 m
Culture T (°C)	37°C	37°C	28–30°C	28–30°C	35°C
Ability to grow in complex ASW medium	Yes	Yes	Yes	Yes	Yes
*n*-alkane substrate tested	Octane, dodecane, hexadecane	Dodecane	Dodecane	Dodecane	Octane, dodecane, hexadecane
Detection of the gene for *n*-alkane oxidation	*alkB*	*alkB*	ND	ND	*cyp*

a Described in Rosario-Passapera et al. (2012).

“ND” denotes no data available.

In all cases, after the incubation was completed, the supernatant, composed of growth media and compounds produced in cellular metabolism, was collected by centrifugation (8000×g, 15 min), extracted three times with ethyl acetate, concentrated, and the products assayed directly by GC-MS. Control experiments in which all the reaction components were added to the media except for the organisms, were also done to control for abiotic substrate oxidation.

### Mass spectrometry

GC-MS analyses were performed on an Agilent 689N Network GC system with a 6890N Series Injector and 5973N Network Mass selective detector with a HP-5MS crosslinked 5% PH ME Siloxane capillary column (dimensions of 30 m × 0.25 mm × 0.25 μm). Various methods were utilized, but generally the injection temperature was 225°C, and the initial oven temperature was 50°C. Both split and splitless injections were done to optimize peak shape and product detection respectively. The typical GC method ramped the oven to a final temperature of 220°C with a ramp rate of 10°C/min. Authentic products were synthesized and their retention times and fragmentation patterns compared to those of the identified peaks in the GC-MS spectra.

### DNA isolation, PCR and phylogenetic analyses

Genomic DNA was extracted from cells collected by centrifugation using the UltraClean™ Microbial DNA isolation kit, according to the manufacturer's instructions (MoBio Laboratories). The 16S rRNA, *alkB*, and *cyp153* genes were selectively amplified from the genomic DNA by PCR, sequenced and subjected to phylogenetic analyses as described previously (Vetriani et al., 2004; Crespo-Medina et al., 2009; Pérez-Rodríguez et al., 2010; Rosario-Passapera et al., 2012).

## Results

### Characterization of hydrocarbonoclastic bacteria

**Figure 1:**
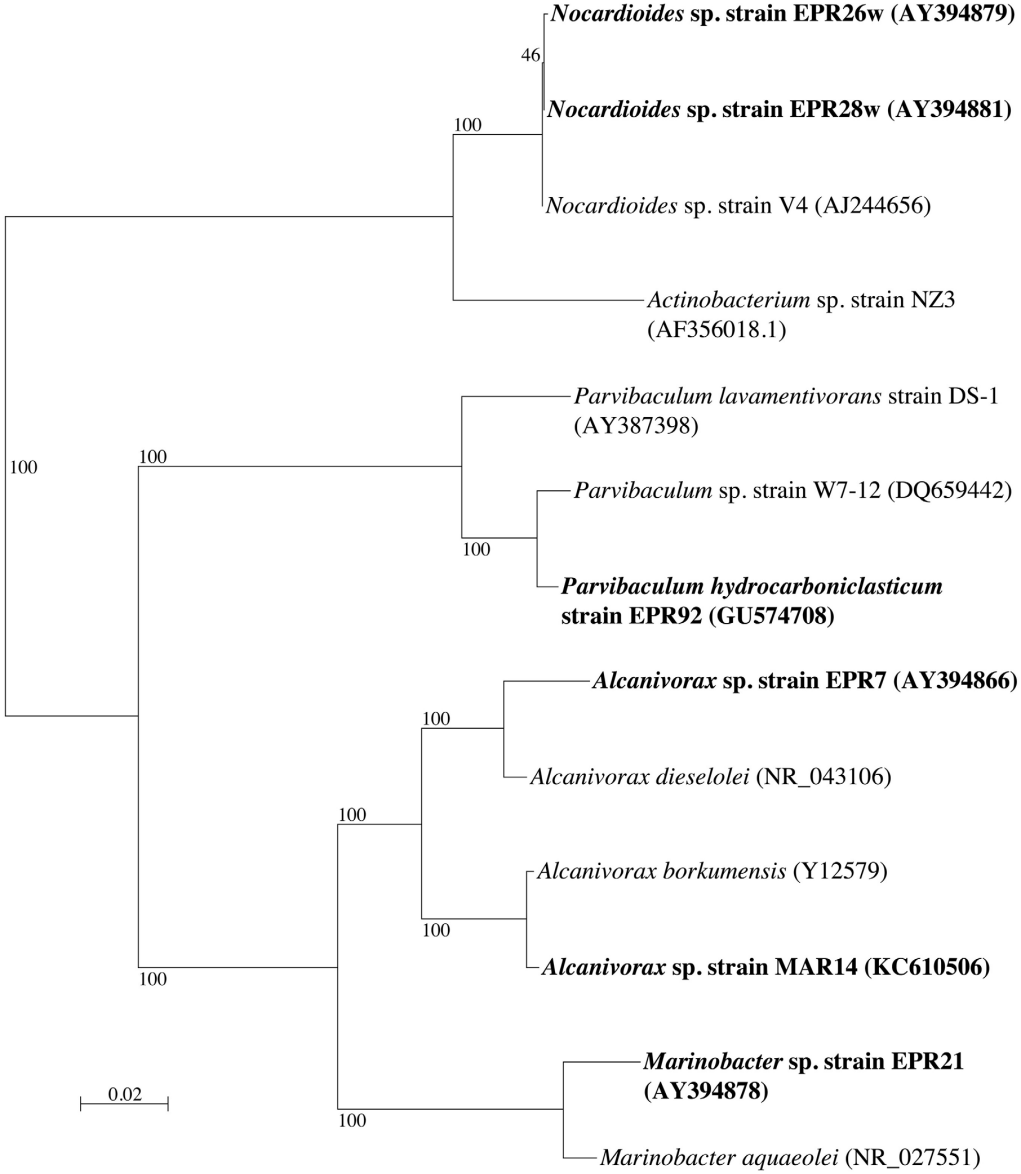
**Neighbor-joining phylogenetic tree inferred from 16S rRNA gene sequences, showing the position of the six deep-sea hydrothermal vent strains (in boldface) used in this study.** The tree was constructed using Phylo_Win. Bootstrap values based on 100 replications are shown as percentages at branch nodes. Bar indicates 2% estimated substitution. Accession numbers for all strains are given in parentheses.

**Figure 2:**
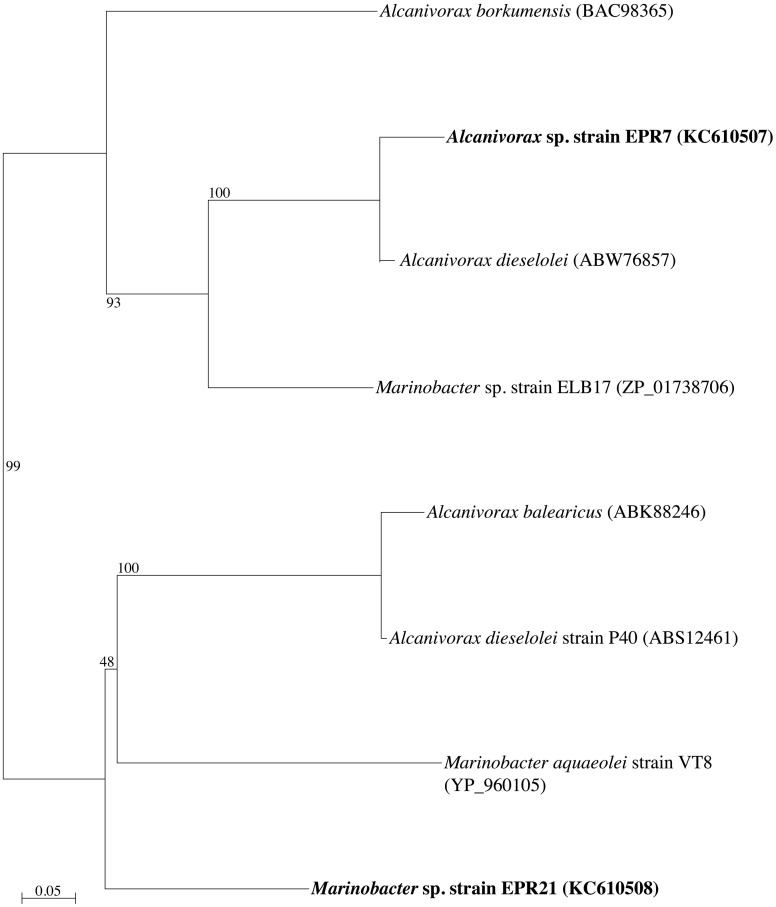
**Neighbor-joining phylogenetic tree inferred from amino acid sequences deduced from the nucleotide sequence of a fragment of the *alkB* gene (encoding for the alkane hydroxylase), showing the position of *Alcanivorax* sp. strain EPR7 and *Marinobacter* sp. strain EPR21 (in boldface).** The tree was constructed using Phylo_Win. Bootstrap values based on 100 replications are shown as percentages at branch nodes. Bar indicates 5% estimated substitutions.

Phylogenetic analysis of the 16S rRNA gene of the six alkane-oxidizing bacteria used in this study placed them in three different taxonomic groups. Figure 1 shows a 16S rRNA gene-based tree illustrating these relationships. Strains EPR 7, MAR14, and EPR21 are *Gammaproteobacteria* of the genus *Alcanivorax* (EPR7 and MAR14) and *Marinobacter* (EPR21), EPR 26w and 28w are *Actinobacteria* related to the *Nocardioides* and strain EPR92 is an *Alphaproteobacterium* that has been recently described as a new species of the genus *Parvibaculum*, *P. hydrocarbonoclasticum* (Rosario-Passapera et al., 2012). Table 1 summarizes the main characteristics of the bacteria used in this study. PCR amplification of the genes putatively involved in the oxidation of *n*-alkanes in three of the six strains used in this study showed that *Alcanivorax* sp. strain EPR7 and *Marinobacter* sp. strain EPR21 encoded for the AlkB alkane hydroxylase, while *P. hydrocarbonoclasticum* strain EPR92 encoded for CYP (Rosario-Passapera et al., 2012). Figure 2 shows a phylogenetic tree inferred from AlkB amino acid sequences that shows the position of the alkane hydroxylase from strains EPR7 and EPR21 relative to closely related enzymes.

### Mechanistic studies

**Figure 3:**
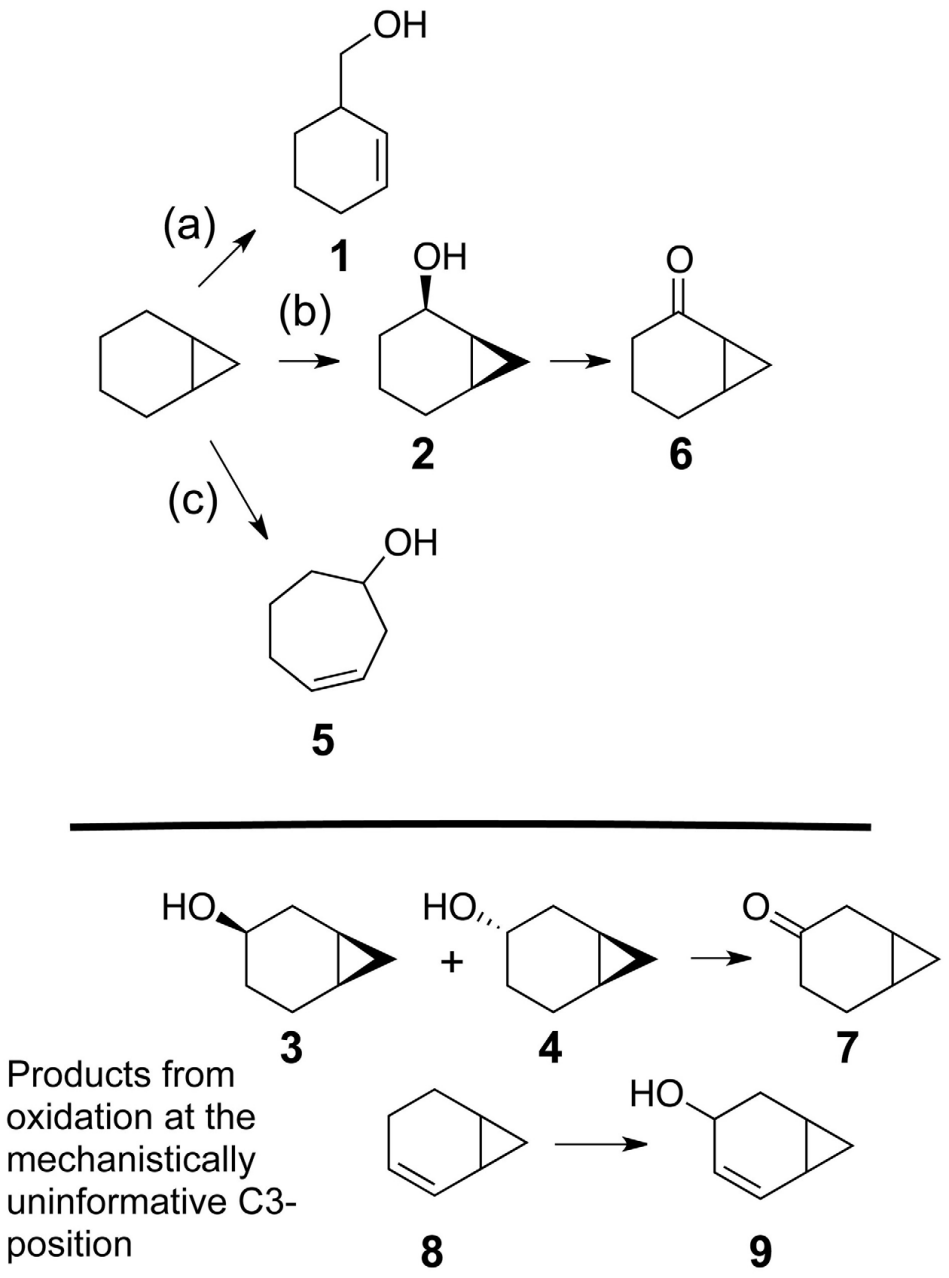
**Possible products from the oxidation of norcarane. (a)** radical pathway **(b)** insertion pathway or pathway of short lived radical **(c)** cationic pathway. **1–9** represent specific compounds that can be formed from the oxidation of norcarane.

**Figure 4:**
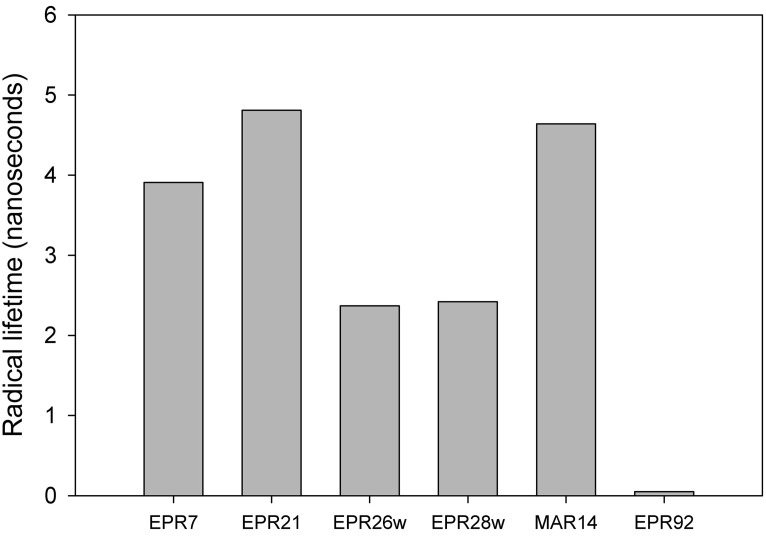
**Measured radical lifetimes for hydrothermal vent isolates EPR7, EPR21, EPR26w, EPR28w, MAR14, and EPR 92 using norcarane as the radical clock substrate**.

**Figure 5:**
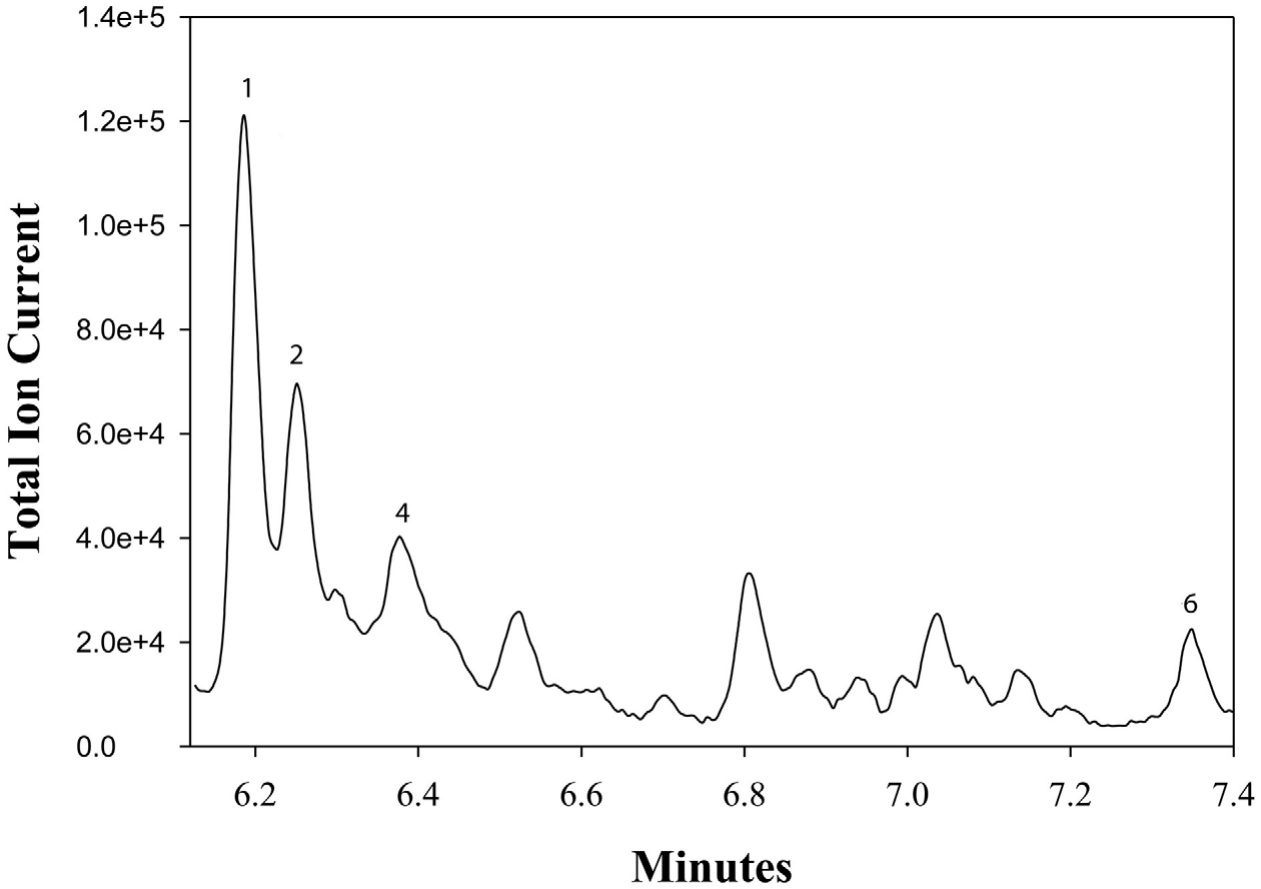
**A chromatogram of norcarane hydroxylation products, as metabolized by strain EPR21.** Norcarane was introduced in the vapor phase, and the cells were then incubated for 10 h. Products are identified in Figure 3.

**Figure 6:**
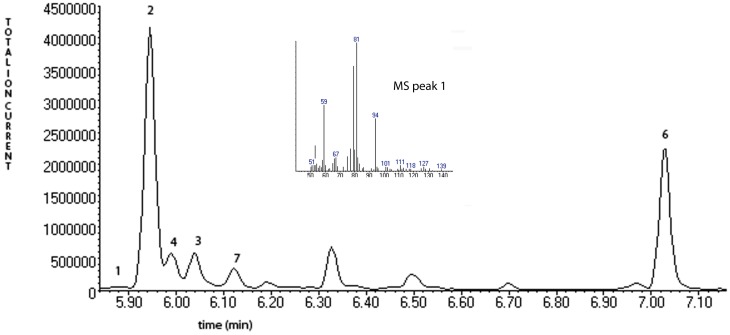
**A chromatogram of norcarane hydroxylation products, as generated by strain EPR92.** Products are identified in Figure 3. The inset shows the fragmentation pattern for peak 3, characteristic of the ring-opened radical product (**1** in Figure 3).

Figure 3 describes products formed during enzymatic oxidation of norcarane, which are used here to characterize alkane oxidation mechanisms in strains EPR7, EPR21, EPR26w, EPR28w, MAR14, and EPR92. Figure 4 shows the calculated radical lifetime determined from the oxidation of norcarane by these strains, demonstrating that EPR7, 21, 26w, 28w, and MAR14 are all using AlkB-like enzymes to oxidize alkanes. This is clear from the substantial amount of rearranged alcohols detected with these assays (radical lifetimes of 3.9, 4.8, 2.4, 2.4, and 4.6 ns respectively), which is the signature for the AlkB enzyme (Austin et al., 2000, 2008; Bertrand et al., 2005; Cooper et al., 2012; Naing et al., 2013). DNA analysis, described in section “Characterization of Hydrocarbonoclastic Bacteria,” confirms that EPR7 and EPR21 contain an *alkB* gene. EPR92, in contrast, is using a CYP-like enzyme to oxidize alkanes, as evidenced by the minuscule amount of rearranged alcohols, which leads to a very short radical lifetime (50 ps), characteristic of all CYPs that have been examined (Austin et al., 2006). DNA analysis, described above, confirms that EPR92 has a *cyp* gene. Chromatograms for GC-MS analysis of EPR92 and EPR21-catalyzed oxidation of norcarane are provided in Figures 5 and 6. The insert in Figure 6 shows the fragmentation pattern for the radical ring-opened product (structure 1 in Figure 3).

## Discussion

Our study reveals two main findings. First, we describe some of the first medium-chain alkane-oxidizing mesophilic bacterial isolates from hydrothermal vent environments, suggesting that alkane oxidation could be an important component of microbial metabolism in diffuse flow vent environments. Second, we identify the enzymes responsible for this activity as well-characterized AlkB and CYP-like enzymes, suggesting that the mechanisms responsible for medium-chain alkane oxidation in the surface ocean and other environments are also active in extreme environments.

While the occurrence of petroleum hydrocarbons in deep-sea geothermal environments has been documented for over 20 years (e.g., Brault et al., 1988; Didyk and Simoneit, 1989), most of the studies on microbial oxidation of hydrocarbons in these environments are focused on short-chain alkanes (C_1_–C_4_; Wankel et al., 2010, 2012). Hence, our knowledge of the taxonomic, physiological, and metabolic diversity of mid-chain (C_6_–C_16_) or long-chain (>C_16_) alkane-oxidizing bacteria in deep-sea geothermal environments remains very limited. An early study of microbial oxidation of hexadecane and naphthalene by bacteria isolated from deep-sea hydrothermal sediments revealed activity under aerobic and mesophilic conditions (Bazylinski et al., 1989). However, neither the organisms, nor the enzymes, responsible for the oxidation of these hydrocarbons were identified in this study. More recently, the description of two mesophilic *Proteobacteria* isolated from deep-sea hydrothermal vents and capable of growth on *n*-alkanes as their sole carbon source was reported (Crespo-Medina et al., 2009; Rosario-Passapera et al., 2012). Furthermore, aerobic, hydrocarbonoclastic bacteria were isolated from deep-sea sediments collected in the Atlantic Ocean (depth: 3542 m) and in the Mediterranean Sea (depth: 2400 m). The isolates obtained in these studies were related to known genera of marine bacteria, including *Alcanivorax, Marinobacter*, and *Halomonas* spp., among others (Wang et al., 2008; Tapilatu et al., 2010). Finally, two recent culture-independent surveys of the genes encoding for the alkane hydroxylase, *alkb*, revealed the presence of these genes in bacteria from sediments collected from depths of 100–400 m and 5724 m, respectively (Xu et al., 2008; Wasmund et al., 2009). However, to our knowledge, our study is the first to probe mechanisms of alkane-oxidizing metalloenzymes from aerobic, hydrocarbonoclastic bacteria from deep-sea hydrothermal vents.

Here we also report the identification and mechanisms of the alkane-hydroxylases from of six strains of aerobic, mesophilic, hydrocarbonoclastic bacteria isolated from deep-sea hydrothermal vents. The six vent organisms belong to the genera *Alcanivorax* (EPR7 and MAR14), *Marinobacter*, (EPR21), *Nocardioides* (EPR26w and 28w) and the previously described *P. hydrocarbonoclasticum* EPR92 (Rosario-Passapera et al., 2012). We demonstrate that both AlkB and CYP are functional in these organisms, which is consistent with their taxonomic assignments and previous work describing these enzyme classes in bacteria from other environments. The apparent trend toward enzyme redundancy in each class of alkane-oxidizing enzymes, including this study, is notable. Implications for this redundancy are discussed below.

While different classes of enzymes exist that oxidize methane, propane and butane, medium chain (C_5_–C_22_) alkanes, and long chain alkanes, there seems to be redundancy in most of these classes. For example, both pMMO and sMMO oxidize methane, there are both particulate and soluble propane and bMO, AlkB, and CYP both oxidize medium chain alkanes, and LadA and AlmA both oxidize long chain alkanes (Austin and Groves, 2011). Some hydrocarbonoclastic organisms appear to express only one kind of alkane-oxidizing enzyme, although they may have multiple genes encoding different isozymes of the same enzyme that could enable them to oxidize alkanes of different chain lengths (Whyte et al., 2002; Van Beilen et al., 2006; Lo Piccolo et al., 2011; Nie et al., 2013). Other organisms contain multiple different alkane-oxidizing genes (e.g., both *cyp* and *alkb*) and may express them simultaneously (Ishikawa et al., 2004; Schneiker et al., 2006; Hakemian and Rosenzweig, 2007; Liu et al., 2011; Lo Piccolo et al., 2011; Nie et al., 2013). The reason for the redundancy in microbial alkane oxidizing enzymes is not clear, nor is it clear what factors control their expression.

Possible factors that contribute to this redundancy include metal availability and subcellular enzyme localizations. Many of these alkane-degrading enzymes require metals for catalysis and their expression can be a function of metal availability. sMMO, for example, is only expressed under copper-limiting conditions (Hakemian and Rosenzweig, 2007). Butane and propane monooxygenases come in both a soluble diiron form and a particulate copper-containing form as well (Austin and Groves, 2011). AlkB and CYP are both iron containing enzymes, but AlkB requires two iron atoms for activity while CYP only one (Shanklin et al., 1997; Van Beilen et al., 2006). Additionally, in most cases of alkane oxidizing enzyme redundancy, one enzyme is a membrane-spanning enzyme (pMMO, particulate butane and propane monooxygenase, AlkB) while the other enzyme in the class is soluble (sMMO, soluble butane and propane monooxygeanse, CYP). It seems possible that there is an as of yet undescribed functional reason to maintain a membrane bound versus soluble enzyme or vice versa.

Since all of the organisms studied here are mesophilic alkane degraders isolated in the same manner from similar environments, it suggests they may fill similar ecological roles. Yet our study shows that they use two distinct enzymes to accomplish the same task. Under the experimental conditions employed here, only EPR92 expresses CYP (consistent with the presence of only this enzyme in its genome and in the complete genome sequence of its close relative, *P. lavamentivorans*), while all of the other organisms express AlkB-like hydroxylases (regardless of whether they have multiple enzyme systems, which is not yet known). Whether the iron quota differences that would result from expressing a diiron protein (AlkB) vs. a single-iron heme protein (CYP) is significant to a hydrocarbonoclastic organism in low iron/high alkane environments is not clear. In the case of deep-sea vents, however, iron should not be a limiting factor since vent plumes appear to be a source of iron to the global ocean (Noble et al., 2012). Slightly different substrate ranges might explain the coexistence of both enzymes in the same environment, as EPR92 grew well on octane while the other organisms were all grown on dodecane, although a detailed characterization of the substrate ranges of the specific enzymes in these organisms has not been done. CYP and AlkB enzymes are known to have very similar substrate ranges. However, AlkB and CYP enzyme classes have multiple isoforms, each with the ability to oxidize only a limited range of alkanes (Whyte et al., 2002; Van Beilen et al., 2005; Naing et al., 2013; Nie et al., 2013).

**Figure 7:**
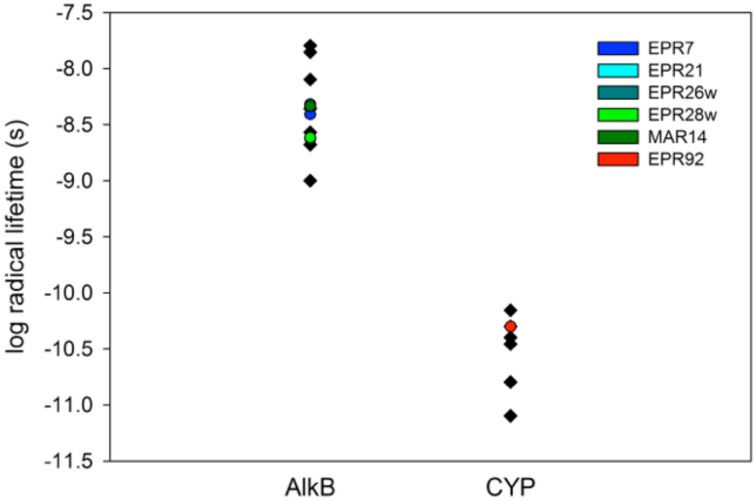
**Log of the radical lifetime for a series of AlkB and CYP-containing organisms.** Data points given in black were previously described (Rozhkova-Novosad et al., 2007). Entries marked in color are from this work. EPR7, 21, 26w, 28w, and MAR14 clearly cluster with AlkB containing organisms while EPR92 displays a CYP-like radical lifetime.

We studied the alkane-oxidizing behavior of six organisms isolated from deep-sea vents and did not find evidence for a novel alkane oxygenase reaction mechanism, since, as shown in Figure 7, all six isolates generated norcarane profiles that were entirely consistent with expression of either AlkB or CYP. We thus report that the two enzymes thought to be responsible for catalyzing the hydroxylation of medium-chain alkanes in surface waters, AlkB and CYP (Wang et al., 2010a), are likely employed for alkane oxidation in organisms isolated from deep-sea vents. Since the organisms studied in this report are closely related to strains isolated from different marine environments (Figure 1), it is not surprising that they encode functionally similar enzymes to those isolated from other environments. Given that it is theoretically possible that unknown alkane hydroxylases may function with similar reaction mechanisms to AlkB or CYP, these results alone do not entirely rule out the possibility of an unidentified alkane hydroxylase in these organisms. However, considering the characteristic patterns observed here (Figure 7), the PCR confirmation, and the ubiquity of similar organisms in other environments where AlkB and CYP are known to be abundant, we find this an extremely unlikely possibility.

This study supports the notion that alkane oxidation may be an important metabolism in diffuse flow vent environments and that this alkane oxidation is supported, at least in part, by well characterized iron-containing metalloenzymes. These are among the first documented cases of alkane-oxidizing enzymes from deep-sea hydrothermal vents. It remains unclear whether the deep-sea vent alkane hydroxylation via AlkB and CYP reported here is widespread. Future efforts should confirm such activity *in situ*. Environmental transcriptomics and proteomics studies coupled with *in-situ* activity assays would offer such evidence. This, coupled with geochemical profiles of alkane availability in vent environments, would additionally allow for an evaluation of what percentage of vent microbial activity is supported by heterotrophic growth on alkanes, and important consideration since microbial life in vent environments support rich, unique ecosystems. Given that alkane oxidizing organisms encoding AlkB and CYP enzymes have been identified in oceanic surface waters (Wang et al., 2010a,b), it remains to be seen whether alkane oxidation is of elevated importance in vent environments over deep water or surface water marine sites. In order to evaluate this, future studies should compare hydrocarbon availability and the diversity and abundance of known metal- and flavin-containing alkane hydroxylases between deep water, vent plume, vent sediment and shallow marine water.

### Conflict of interest statement

The authors declare that the research was conducted in the absence of any commercial or financial relationships that could be construed as a potential conflict of interest.
